# The Alkaloid Gelsemine Reduces Aβ Peptide Toxicity by Targeting Transglutaminase Type 2 Enzyme

**DOI:** 10.3390/plants14101556

**Published:** 2025-05-21

**Authors:** Jessica Panes-Fernández, Ana M. Marileo, Nicole Espinoza-Rubilar, Macarena E. Meza, Bernardita A. Salgado-Martínez, Krishna Gaete-Riquelme, Gustavo Moraga-Cid, Patricio A. Castro, Carlos F. Burgos, Jorge Fuentealba, Gonzalo E. Yévenes

**Affiliations:** 1 Laboratorio de Screening de Compuestos Neuroactivos, Departamento de Fisiología, Facultad de Ciencias Biológicas, Universidad de Concepción, Concepción 4070409, Chile; jpanes@udec.cl (J.P.-F.); nicespinoza@udec.cl (N.E.-R.); macarenameza@udec.cl (M.E.M.); 2 Laboratorio de Neurofarmacología, Departamento de Fisiología, Facultad de Ciencias Biológicas, Universidad de Concepción, Concepción 4070409, Chile; anamarileo@udec.cl (A.M.M.); besalgado2018@udec.cl (B.A.S.-M.); kgaete2019@udec.cl (K.G.-R.); 3 Departamento de Fisiología, Facultad de Ciencias Biológicas, Universidad de Concepción, Concepción 4070409, Chile; gumoraga@udec.cl (G.M.-C.); pacastro@udec.cl (P.A.C.); caburgos@udec.cl (C.F.B.)

**Keywords:** gelsemine, natural alkaloid, Alzheimer’s disease (AD), β-amyloid peptide (Aβ peptide), transglutaminase type 2 (TG2), neuronal activity

## Abstract

Gelsemine, a naturally occurring indole alkaloid derived from plants of the *Gelsemium* species of the Gelsemiaceae family, has been extensively investigated for its neuroprotective and anti-inflammatory properties. Recent studies have demonstrated that gelsemine exerts neuroprotective effects against beta-amyloid (Aβ) oligomers, a key neurotoxic peptide implicated in the pathogenesis of Alzheimer’s disease (AD). However, despite these beneficial effects, the precise molecular targets underlying gelsemine’s neuroprotective actions in AD remain unidentified. Here, we employed a combination of bioinformatic, biochemical, and functional assays in neuronal models to investigate the mechanism of gelsemine’s action in AD cellular models. Our findings indicate that gelsemine inhibits the activity of transglutaminase 2 (TG2), an enzyme involved in protein cross-linking with emerging roles in Aβ aggregation and neurotoxicity. Molecular modeling and biochemical analyses reveal that gelsemine interacts with the TG2 catalytic site, leading to its inhibition. Furthermore, gelsemine modulates the TG2-mediated Aβ aggregation process, thereby attenuating Aβ-induced neurotoxicity and preserving neuronal function. These findings establish TG2 as a previously unrecognized molecular target of gelsemine and underscore the potential of *Gelsemium*-derived alkaloids as neuroprotective agents. The modulation of TG2 activity by natural alkaloids may provide a novel therapeutic approach for mitigating Aβ toxicity and preserving neuronal function in AD.

## 1. Introduction

Traditionally used in Asian folk medicine, alkaloid extracts from *Gelsemium* have been employed to treat persistent pain, anxiety, neuralgia, sciatica, rheumatoid arthritis, and various inflammatory conditions. Gelsemine, the main indole alkaloid from the *Gelsemium* species of the Gelsemiaceae family, has been extensively investigated for its neuroprotective, analgesic, and anti-inflammatory properties, as well as a wide range of additional pharmacological properties [1,2]. Behavioral assays using purified gelsemine have shown analgesic [3,4,5,6], anxiolytic [7,8,9], anti-inflammatory [10], hypnotic [5], and neuroprotective properties in rodents [11], as well as anti-proliferative actions in cellular models of cancer [12].

So far, the molecular mechanisms underlying these therapeutic effects remain undefined. Nevertheless, gelsemine has been shown to modulate the function of several enzymes and ion channels participating in the regulation of neuronal activity. For example, biochemical studies have determined that gelsemine is able to stimulate the activity of 3α-hydroxysteroid oxide-reductase (3α-HSOR) [3,6,13], which is involved in the neurosteroid production in the central nervous system [3,4,6,13,14]. On the other hand, functional studies combined with bioinformatics have demonstrated that glycine and GABA type A receptors, the main inhibitory ion channels of the CNS, are majorly inhibited by gelsemine [15,16]. Interestingly, recent studies have utilized molecular docking and network pharmacology to enhance our understanding of the molecular targets of alkaloids from *Gelsemium elegans*. A potential stable interaction of koumine with the epidermal growth factor receptor (EGFR) has been identified [17], a critical plasma membrane receptor involved in cell proliferation and colorectal cancer [18]; there has also been confirmation of the interactions of gelsemine with the orthosteric sites of glycine and GABA type A receptors [16,19,20]. This evidence suggests that gelsemine, and possibly other Gelsemium alkaloids, interacts with multiple biological targets, influencing cell signaling and neuronal activity. This multi-targeted modulation may underlie gelsemine’s broad beneficial effects observed in both in vitro and in vivo models of human diseases [4,5,6,7,8,11,20,21,22,23,24,25,26].

A study conducted a few years ago demonstrated that gelsemine exhibits significant neuroprotective effects against beta-amyloid (Aβ) oligomers, a key neurotoxic peptide involved in Alzheimer’s disease (AD). At low concentrations (5–10 μg/kg), gelsemine improved cognitive and spatial impairments in Aβ-treated mice [11]. It also reduced neuroinflammation by inhibiting the overactivation of microglia and astrocytes, as indicated by the decreased levels of pro-inflammatory cytokines such as IL-1β, IL-6, and TNF-α [11]. However, despite these promising results, potential molecular targets of gelsemine in neuroprotection and AD remain unidentified.

The accumulation of β-amyloid (Aβ) aggregates is a key factor in neuronal damage in AD, disrupting calcium levels and neurotransmitter balance and leading to synaptic dysfunction [27]. Over the past two decades, the prevention and treatment of Aβ pathology in AD have been hindered by the failure to recognize the physiological role of continuously produced and aggregated nascent Aβ monomers in synaptic processing. Recent findings have demonstrated that transglutaminase 2 (TG2), an enzyme responsible for cross-linking proteins, exacerbates AD by promoting and stabilizing the generation of Aβ aggregates (AβTG2), thereby contributing to the disease’s development [28]. The activity of TG2 is tightly regulated by conformational shifts between its closed (inactive) and open (active) states [29]. Interestingly, chemical inhibitors targeting TG2’s catalytic site have been investigated as potential therapeutic interventions for conditions such as celiac disease and neurological disorders, including AD [28].

Given the growing evidence of TG2’s role in AD [28,30,31,32,33,34] and the neuroprotective effects of gelsemine in AD models [11], we hypothesize that a functional interaction between gelsemine and TG2 may underlie its beneficial actions in AD. Thus, this study aims to determine whether the potential interaction between the TG2 enzyme and gelsemine contributes to their neuroprotective effects against β-amyloid-induced toxicity in cellular models of AD. Through a combination of bioinformatics, biochemical, and functional assays in neurons, we provide data supporting the assertion that gelsemine reduces TG2’s catalytic activity, thereby mitigating Aβ toxicity and preserving neuronal function. Our findings identify the TG2 enzyme as a previously unrecognized molecular target of gelsemine, presenting a novel therapeutic strategy to counteract Aβ toxicity and protect neuronal function in AD.

## 2. Results

### 2.1. In Silico Binding of Gelsemium Alkaloids to TG2

**Figure 1:**
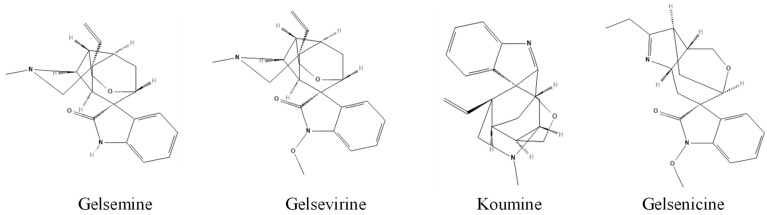
Chemical structures of key indole alkaloids from *Gelsemium*. Structures were obtained from PubChem (CIDs: 5390854 (171384473), Gelsemine; 14217344 (5491636, 125114838), Gelsevirine; 21123652, Gelsenicine; 102004413, Koumine).

**Figure 2:**
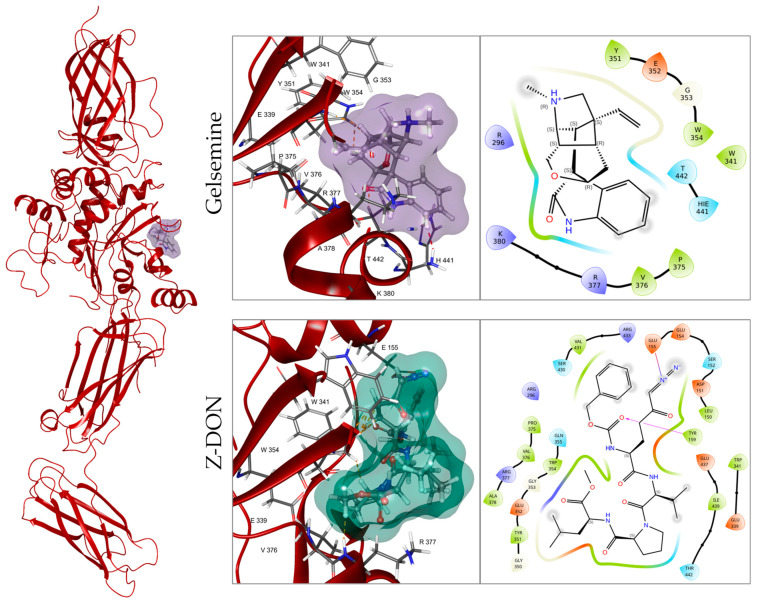
Interaction of gelsemine with the TG2 catalytic site. Left: full TG2 structure (PDB: 2Q3Z) bound to gelsemine (purple). Right: molecular interactions between the TG2 catalytic site (red) and gelsemine (purple) or Z-DON (green). In both cases, hydrophobic (green) and hydrophilic (light blue) interactions contribute to binding stability. However, Z-DON forms a hydrogen bond (purple arrows) between its hydroxyl group and tyrosine residue Y159. Amino acid properties are color-coded: green (hydrophobic), red (negatively charged), blue (positively charged), cyan (polar), and light yellow (glycine).

To assess whether gelsemine and other *Gelsemium* indole alkaloids may interact with TG2, we performed in silico molecular docking using the crystal structure of TG2 as a template (PDB: 2Q3Z) [35]. Due to previous reports suggesting neuroprotective actions [11], we focused these analyses on four indole alkaloids: gelsemine, koumine, gelsevirine, and gelsenicine (Figure 1). Docking scores and binding free energy (ΔG) calculations indicate a favorable interaction between gelsemine (−32.24 kcal/mol) and koumine (−31.35 kcal/mol) with the catalytic region of TG2, while gelsevirine (−22.07 kcal/mol) and gelsenicine (−21.77 kcal/mol) showed less favorable binding affinities (Table 1). Stereoisomeric structures for gelsemine showed similar values. On the other hand, gelsevirine stereoisomers displayed some divergencies in terms of energetic parameters. Interestingly, gelsemine interacts with key residues of TG2, such as W341, Y351, E352, G353, W354, and T442, which are also involved in the binding of the well-known TG2 inhibitor Z-DON (−46.99 kcal/mol) (Table 1), suggesting that both molecules bind within the same region of the enzyme’s catalytic site (Table 1 and Figure 2). Specifically, gelsemine also interacts with H441 and R377, which may provide additional stabilization within the catalytic pocket. Structural comparison with Z-DON revealed a significant overlap in the binding regions, particularly involving residues W341 and T442 (Figure 2). These findings suggest that, while gelsemine shares several binding features with Z-DON, it may modulate TG2 activity differently, possibly as a weaker inhibitor, which could be beneficial as a potential therapeutic target.

### 2.2. In Vitro Gelsemine Inhibition of Recombinant TG2 Activity

**Figure 3:**
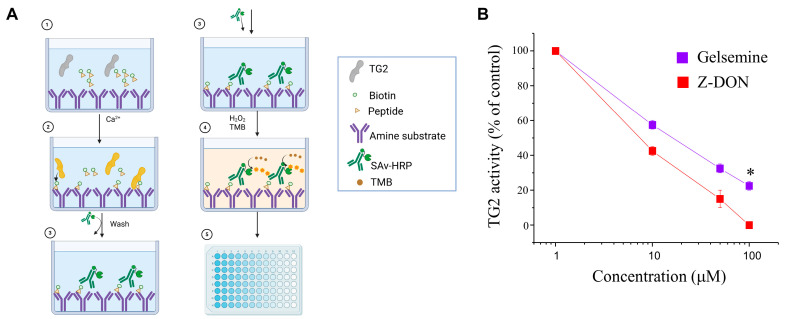
Concentration-dependent inhibition of TG2 activity by gelsemine. (**A**) Schematic representation of the assay used to evaluate the transamidase activity of recombinant TG2. (**B**) Graph depicting the percentage of enzymatic activity relative to the control (% of control) as a function of gelsemine or Z-DON concentration. Both compounds exhibited a concentration-dependent inhibition of TG2 activity. Data points represent the mean percentage of inhibition from three independent assays. * *p* < 0.05, Student’s *t*-test.

Since the catalytic site of TG2 is involved in facilitating the open conformation required for protein aggregation, the gelsemine interaction within this site may prevent such enzymatic activity. To experimentally validate our in silico results, we performed in vitro transamidation assays employing a recombinant version of human TG2 enzyme in the presence of increasing concentrations of gelsemine (1–100 μM). A biotinylated peptide (i.e., biotin-pepT26) functioned as the primary substrate (amine acceptor/acyl donor), whereas an amine donor/acyl acceptor acted as the secondary substrate (Figure 3). Activated TG2 was incubated with gelsemine and the commercial inhibitor Z-DON at increasing concentrations (1–100 μM) for 30 min at 37 °C (see Methods). As expected, gelsemine displayed a concentration-dependent inhibition of the TG2 activity. Z-DON shows a more pronounced reduction in TG2 activity compared to gelsemine. However, Z-DON inhibition was significantly different than gelsemine only at 100 μM.

### 2.3. Altered β-Amyloid Aggregation by Gelsemine in the Presence of TG2

**Figure 4:**
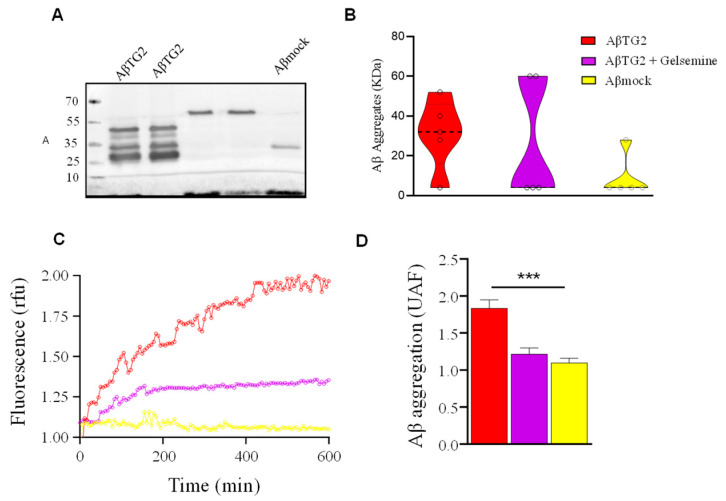
TG2-induced Aβ aggregation was attenuated by gelsemine. (**A**) Representative blot of Aβ aggregates formed by TG2 in the presence or absence of gelsemine (50 μM). (**B**) Quantification of Aβ assemblies under each condition. Gelsemine treatment nearly eliminated detectable Aβ aggregates between 20 and 40 kDa. (**C**) Thioflavin T insertion kinetics for Aβ aggregates induced by TG2, in conditions with or without gelsemine (50 μM). (**D**) Graph showing plateau values at 4 h (*n* = 3, *** *p* < 0.001, ANOVA). Aβ mock was included as a control condition.

Previous studies showed that TG2 promotes the generation and stabilization of Aβ aggregates (so-called Aβ-TG2 aggregates) (9). As these actions were dependent on the catalytic activity of TG2 (9), we next aimed to determine whether the inhibitory TG2 activity detected for gelsemine (Figure 3) is effectively translated on alterations in the Aβ aggregation pattern. We first performed Western blot assays to determine the potential effects of gelsemine on the size of Aβ aggregates (Figure 4A,B). We observed a significant reduction in the number of diverse Aβ aggregate species induced by the catalytic activity of TG2 in the presence of gelsemine. The decrease or absence of bands corresponding to monomers and oligomers, together with the appearance of high molecular weight bands, suggests the formation of “atypical” aggregates. To expand these observations, we performed biochemical experiments measuring the insertion kinetics of Thioflavin T on Aβ-TG2 aggregates (9) in the presence or the absence of gelsemine (Figure 4C,D). These results confirm that the alkaloid was able to decrease the formation of Aβ aggregates in the presence of TG2, suggesting that gelsemine reduces β-sheet formation of Aβ peptide induced by TG2 transamidating activity. Analyses of the dissociation rate values (Kg: Aβ_TG2_: 1.6 × 10^−2^ s^−1^ vs. Aβ_TG2+_Gelsemine: 1.2 × 10^−2^ s^−1^) and the maximum reaction velocity (Vmax: Aβ_TG2_: 1.8 vs. Aβ_TG2+_Gelsemine: 1.4 rfu) suggest that the alkaloid reduces the activity of TG2 by decreasing both the dissociation rate and the maximum reaction capacity. Altogether, these observations suggest that gelsemine possibly does not act as an aggregation process inhibitor, but likely promotes the formation of different types of Aβ protein associations. Therefore, these Aβ-TG2 aggregates formed in the presence of gelsemine may have functional and structural differences in terms of their neuronal toxicity compared to those generated without the alkaloid.

### 2.4. Functional Validation of Gelsemine Neuroprotective Actions in a Neuronal Model of AD

**Figure 5:**
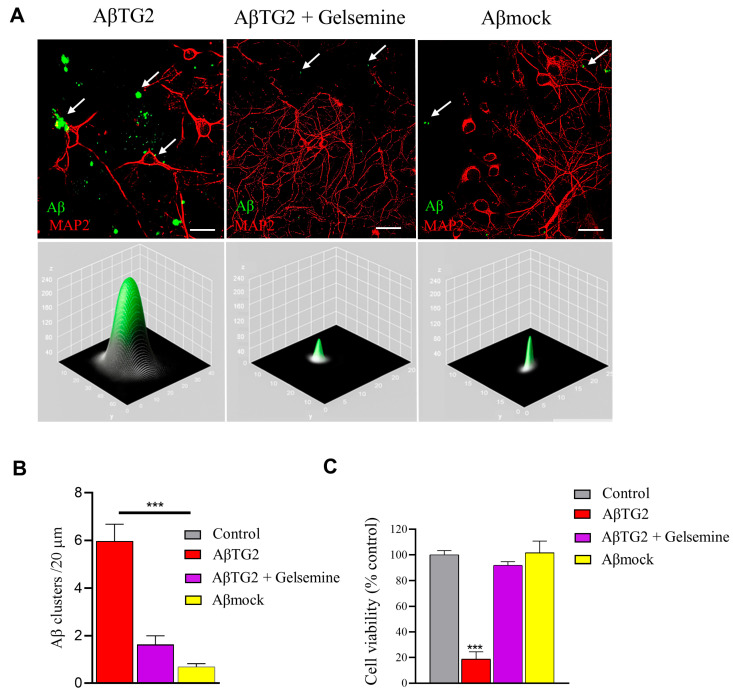
Gelsemine preserves cell viability and reduces Aβ-TG2 aggregate association with hippocampal neurons. (**A**) Immunofluorescence images showing Aβ-TG2 aggregates (green) associated with hippocampal neurons (MAP2, red) in the absence and presence of gelsemine (scale bar: 10 μm). White arrows indicate Aβ deposits near neurons. The lower panel presents a surface plot of representative Aβ aggregates from the images. (**B**) Quantification of Aβ clusters in samples incubated with TG2, under conditions with or without gelsemine. (**C**) Cytotoxicity induced by Aβ aggregates in the absence and presence of gelsemine. *n* = 3–5 assays, *** *p* < 0.001, ANOVA. Assays were performed using 50 μM of the alkaloid.

**Figure 6:**
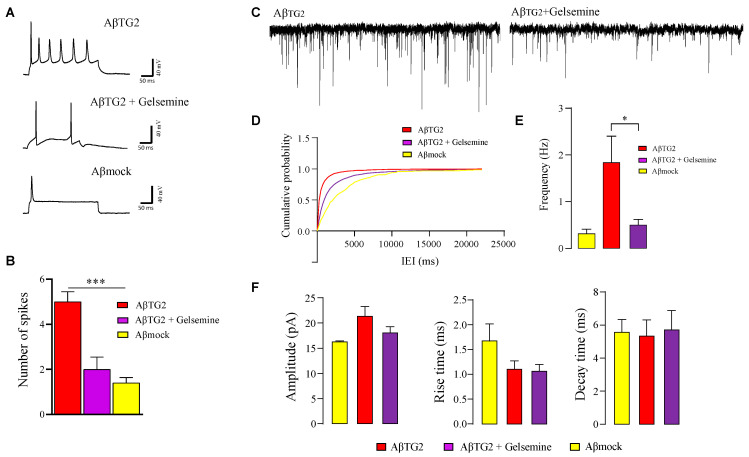
Functional neuroprotective effects of gelsemine in hippocampal neurons exposed to Aβ-TG2 aggregates. (**A**) Representative action potential (AP) firing traces from primary hippocampal neurons treated with Aβ-TG2 aggregates (0.5 µM, 30 min) in the absence or presence of gelsemine (50 µM). (**B**) Quantification of AP spike number. Data are expressed as mean ± SEM (*n* = 7–8). *** *p* < 0.001, ANOVA. (**C**) Representative current traces showing miniature excitatory postsynaptic currents (mEPSCs) in neurons treated with Aβ-TG2 aggregates, with or without gelsemine (50 µM). (**D**) Cumulative probability distribution of mEPSC inter-event intervals in neurons treated with Aβ-TG2. The distribution was significantly altered by gelsemine (*p* < 0.0001, Kolmogorov–Smirnov test) and was comparable to that observed under Aβ-mock conditions. (**E**) Bar graph showing the effect of gelsemine (50 µM) on mEPSC frequency in neurons treated with Aβ-TG2. Gelsemine significantly restored mEPSC frequency. * *p* < 0.05, one-way ANOVA followed by Tukey’s post hoc test. (**F**) Bar plots summarizing the effects of gelsemine on mEPSC amplitude (left) and excitatory current kinetics (rise time and decay time). No significant differences were observed (one-way ANOVA followed by Tukey’s post hoc test).

The findings described above highlight the inhibitory actions of gelsemine on the TG2-mediated Aβ aggregation process. These data suggest that gelsemine may attenuate the TG2-mediated amyloidogenic process, potentially reducing the formation of neurotoxic Aβ species. To assess this hypothesis experimentally, we evaluated the neuroprotective actions of gelsemine on the neurotoxic effects induced by Aβ_TG2_ in a cellular model of AD toxicity based on cultured mouse hippocampal neurons (REFS) (Figure 5 and Figure 6). Immunofluorescence experiments revealed that gelsemine significantly reduced the association of Aβ_TG2_ aggregates with hippocampal neurons (Figure 5A,B). Additionally, cell viability assays indicated that gelsemine significantly decreased the cell death of hippocampal neurons treated with Aβ_TG2_ (Figure 5C).

To assess whether Aβ_TG2_ aggregates formed in the presence of gelsemine induced alterations on neuronal function, we performed electrophysiological recordings of hippocampal neurons (Figure 6). We first assessed neuronal excitability by measuring action potential firing in neurons exposed to Aβ_TG2_ aggregates for 30 min. As previously shown (9), the Aβ_TG2_ aggregates promote an increased action potential firing, reflecting an enhanced excitability (Figure 6A,B). Aβ_TG2_ aggregates prepared in the presence of gelsemine (50μM) preserved the action potential firing within control values (Aβ-mock condition). We next analyzed the impact of Aβ_TG2_ aggregates and gelsemine on the spontaneous excitatory synaptic activity of hippocampal cultures (Figure 6C–F). In line with previous reports (9), incubation with Aβ_TG2_ aggregates (30 min) significantly enhanced the frequency of miniature spontaneous excitatory postsynaptic currents (mEPSCs) without altering the amplitude or the current kinetics (9). On the contrary, Aβ_TG2_ aggregates made in the presence of gelsemine (50μM) were not able to alter the frequency of mEPSCs, preserving a normalized cumulative probability of release. The alkaloid addition preserved the synaptic parameters obtained under control conditions (i.e., Aβ_mock_) despite the presence of Aβ_TG2_, suggesting an effective functional neuroprotective action on the synaptic level.

## 3. Discussion

*Gelsemium* alkaloids have long been used in traditional medicine and exhibit diverse pharmacological effects, particularly in the nervous system. Gelsemine, a prominent indole alkaloid found in *Gelsemium* plants, has demonstrated analgesic [3,4,5,6], anxiolytic [7,8,9], anti-inflammatory [10], and neuroprotective properties [11]. While the precise molecular mechanisms underlying these beneficial effects remain unclear, several experimental studies have identified key molecular targets for gelsemine, including the 3α-hydroxysteroid oxide-reductase (3α-HSOR) enzyme [3,4,6,13,14] and inhibitory glycine and GABA type A receptors [3,15,16,19]. In this study, we demonstrate that gelsemine inhibits the activity of the TG2 enzyme, likely through interactions with its catalytic site. Furthermore, we present data showing that gelsemine-mediated TG2 inhibition modifies the aggregation process of the Aβ peptide, significantly reducing its neurotoxicity at the cellular level.

Gelsemine has been suggested to exert neuroprotective effects in Alzheimer’s disease (AD), as demonstrated by Chen et al., who reported a 40% reduction in plaque formation in Aβ oligomer-treated mice [11]. Certain gelsemine analogs, such as rhynchophylline [36] and isorhynchophylline [37], have also shown neuroprotective and anti-AD properties. Notably, the effective doses of gelsemine (5–10 μg/kg) [11] required to mitigate cognitive impairment were significantly lower than those for rhynchophylline (50 mg/kg) [36] and isorhynchophylline (20 mg/kg) [37], suggesting that gelsemine is the most potent anti-AD compound among its structural analogs [11]. It has been proposed that gelsemine and its analog compounds may target the same molecular pathway, with gelsemine showing stronger actions. Our findings reinforce this evidence by demonstrating that, at the cellular level, gelsemine mitigates the toxic effects of the Aβ peptide on neuronal viability and function. However, the precise molecular target of gelsemine’s neuroprotective effects, particularly its role in inhibiting Aβ aggregation, remains unidentified and continues to be an area of active research. In this context, our work provides a plausible mechanism underlying the neuroprotective actions of gelsemine, which may also extend to other *Gelsemium* alkaloids.

Recent research suggests that transglutaminase 2 (TG2) plays a critical role in Aβ peptide aggregation in Alzheimer’s disease (AD), contributing to the neurotoxic effects associated with Aβ accumulation [28,29,30,32,33,34]. Specifically, TG2 transamidating activity can transiently enhance synaptic activity and excitability before ultimately leading to neuronal death [38]. Previous studies have shown that small peptides designed to inhibit TG2 can reduce β-amyloid aggregation and mitigate its toxic effects [28]. Notably, Z-DON (Z-ZON-Val-Pro-Leu-OMe), a potent and irreversible TG2 inhibitor [39,40], has demonstrated promising results; however, its toxicity and long-term in vivo effects remain poorly understood. TG2 inhibition may also disrupt essential physiological processes, including extracellular matrix regulation, cell adhesion, and tissue repair [41]. Given the potential adverse effects of irreversible TG2 inhibition, alternative pharmacological strategies are needed. Our in silico analyses indicate that several indole *Gelsemium* alkaloids, particularly gelsemine and gelsevirine, interact favorably with the TG2 catalytic site, exhibiting energetic parameters comparable to Z-DON. Although the reversibility of gelsemine-mediated TG2 inhibition remains untested, our findings support further investigation into *Gelsemium* alkaloids as potential TG2 modulators with fewer adverse effects.

Our results suggest that gelsemine interacts with the catalytic core of TG2, attenuating Aβ-induced neurotoxicity. TG2 activity is closely linked to Aβ aggregation and excitotoxicity, and its inhibition may enhance neuronal resilience by reducing toxic oligomer formation and preventing ion dyshomeostasis. Specifically, gelsemine’s inhibition of TG2’s transamidating activity mitigates Aβ nucleation, promoting the formation of less toxic species and reducing the burden of highly neurotoxic aggregates. While previous studies, such as [11], have demonstrated the role of TG2 in cognitive dysfunction associated with Aβ pathology, our findings provide a mechanistic perspective at the cellular level. Unlike their behavioral-focused approach, we reveal that direct modulation of TG2 activity by gelsemine prevents synaptic impairment, likely by stabilizing neuronal homeostasis and limiting aberrant protein cross-linking. This distinction underscores a potential therapeutic avenue targeting TG2 as a molecular modulator of Aβ toxicity, rather than solely addressing downstream cognitive deficits. Further structural and functional analyses are needed to clarify the precise molecular interactions between gelsemine and TG2, as well as their broader impact on Aβ aggregation and neuroinflammation. Our results indicate that gelsemine interacts with the catalytic site of TG2, primarily through hydrophobic contacts, which are further stabilized by hydrogen bonding. These molecular interactions suggest a functional inhibition of TG2, preventing its access to the β-peptide during the nucleation phase. This proposed mechanism is corroborated by the observed decrease in amyloid aggregation at neuronal membranes. Taken together, these findings support the hypothesis that gelsemine interferes with TG2-mediated β-sheet formation, thereby modulating the conformational dynamics of Aβ during the seeding process and potentially destabilizing its most toxic oligomeric species. Understanding these mechanisms could provide a more refined strategy for mitigating Aβ-induced neurotoxicity, potentially paving the way for novel neuroprotective interventions.

## 4. Conclusions

Our findings demonstrate that gelsemine inhibits the activity of transglutaminase 2 (TG2), an enzyme increasingly recognized for its role in Aβ aggregation and neurotoxicity. Through molecular modeling and biochemical analyses, we show that gelsemine binds to the catalytic site of TG2, likely via hydrophobic interactions stabilized by hydrogen bonds, leading to functional inhibition of the enzyme. This interaction may disrupt TG2-mediated β-sheet formation, hinder Aβ nucleation and aggregation, and lead to a less compact, potentially less toxic form of Aβ. Consequently, gelsemine preserves neuronal viability and synaptic function, supporting its role as a neuroprotective agent. These results position TG2 as a previously unrecognized molecular target of gelsemine and suggest that other *Gelsemium*-derived alkaloids may represent a novel therapeutic avenue for mitigating Aβ-induced toxicity in AD. Furthermore, the ability of gelsemine to modulate the conformational dynamics of Aβ during the seeding process highlights the broader potential of natural alkaloids to interfere with pathogenic peptide aggregation. Future studies exploring the interactions of additional *Gelsemium* alkaloids with TG2 may yield new candidates for targeting protein cross-linking pathways implicated in neurodegeneration.

## 5. Materials and Methods

Chemicals. Gelsemine hydrochloride (with a purity greater than 99%) was sourced from ChemFaces (Wuhan, China). Other chemicals were obtained from Tocris (Bristol, UK), Hello-Bio (Bristol, UK), Sigma-Aldrich (St. Louis, MO, USA), and AK Scientific (Union City, CA, USA).

In Vitro Aβ Aggregation. Aβ species aggregated by TG2 were prepared as previously published. [28]. Briefly, Aβ1–40 peptide was dissolved in DMSO and then resuspended in PBS at a concentration of 80 μM, reaching a final concentration of 0.5 μM. To study the impact of TG2 on Aβ aggregation, recombinant TG2 (1 μg/mL, equivalent to 0.1 U/mL) was added to a buffer containing CaCl_2_ and was incubated for 30 min at 37 °C.

Primary Hippocampal Cultures. Primary embryonic hippocampal cultures were prepared following a previously published method [16]. Briefly, primary cultures (E18) from C57BL/J6 mice were prepared following the NIH and Universidad de Concepción ethical guidelines. The mice were deeply anesthetized with CO_2_ before cervical dislocation. The cells were plated at 320,000 cells/mL on poly-L-lysine-coated coverslips and maintained at 37 °C with 5% CO_2_.

Thioflavin T Binding Assay. Aβ aggregation was evaluated in a 96-well plate using 20 µM Thioflavin T (ThT). Fluorescence measurements of the ThT-Aβ complex (excitation: 440 nm, emission: 485 nm) monitored the aggregation of Aβ1–40 and TG2 incubated in DPBS buffer at 37 °C for 24 h. Curve fitting for Aβ (1–40) without lag phase was performed using the equation y(t) = a [1 − exp(−kt)].s

In silico Analysis. Protein-ligand docking was performed using the TG2 structures obtained from the Protein Data Bank (PDB ID: 2Q3Z) [35], Strop P, Brunger AT, Khosla C (2007). Prior to docking simulations, the protein structure was prepared using Maestro’s v2022-4 Protein Preparation Workflow tool. This process included the addition of hydrogens, the optimization of H-bond assignments, the determination of protonation states at pH 7 ± 0.2 and filling in missing side chains with Prime. Similarly, the structures of gelsemine, gelsenicine, gelsevirine, koumin and Z-DON were retrieved from the PubChem database (CID: 5390854, 21123652, 102004482, 102004413, 171378763) and prepared using LigPrep to generate ionization states at pH 7 ± 0.2 and possible conformations for each alkaloid (Schrödinger, LLC, New York, NY, USA, 2022). All site-directed docking calculations were performed using Glide (Schrödinger, LLC, New York, NY, NY, USA, 2022) with a grid centered on the catalytic site of protein transglutaminase 2. Predictions were performed employing the extra-precision (XP) configuration with post-docking minimization involving 10 poses per ligand, from which the best pose was selected to represent each protein–ligand complex. The analysis of the complexes covered structural and energetic parameters summarized in the docking score values.

TG2 Enzymatic Activity. Transglutaminase activity was assessed following the protocol previously described [28]. Briefly, microtiter plates (96 wells) with modified terminal γ-carboxyl groups were utilized to measure transglutaminase enzymatic activity using a colorimetric assay (Novus Biologicals, #NBP1-37008, Centennial, CO, USA), based on the protocol described [28]. In brief, samples were incubated with 50 μL of Reaction Buffer (BiotinpepT26/CaCl_2_). Gelsemine and the commercial inhibitor Z-DON were added at increasing concentrations (1–100 μM) for 30 min at 37 °C. Following this, the plates were washed with Tris-HCl and incubated with 100 μL of HRP (1:2000) for 15 min at room temperature. Then, 100 μL of 0.01% H_2_O_2_ and TMB were added for detection. The reaction was stopped by adding 2NH_2_SO_4_, and absorbance was recorded using a Microplate Reader (NOVOstar Labtech, BMG Labtech, Germany) at 450 nm. TG2 activity was calculated and expressed as mU/µg of protein.

Electrophysiology. The whole-cell patch clamp technique was utilized as previously described [16,28]. The whole-cell patch clamp technique was used with an Axopatch 200B amplifier. Miniature post-synaptic currents were isolated using TTX (50 nM) and bicuculline (5 μM). In current-clamp mode, membrane potential (Vm) and action potentials (APs) were recorded with current pulses ranging from −300 pA to +275 pA for 300 ms. The data were analyzed with Clampfit 10.1, focusing on miniature current and AP parameters.

Immunocytochemistry. Based on our previously published method [42], the cells were fixed with 4% paraformaldehyde, permeabilized with 0.1% Triton X-100, and blocked with 10% horse serum. They were incubated for 1 h with primary antibodies (anti-MAP2, anti-Aβ, all at 1:200), followed by 45 min with Cy3 or Alexa Fluor 488 secondary antibodies. Finally, the cells were stained with 300 nM DAPI and mounted with DAKO media.

Western Blot. To evaluate gelsemine’s effect on Aβ1–40 aggregation induced by Tg2, SDS-PAGE (10%) was performed using the protocol published previously [28]. The samples were loaded (20 µL each) and run at 100 V for 120 min and then transferred to nitrocellulose membranes. After blocking with 5% non-fat milk, the membranes were incubated with primary (Anti-Aβ, 1:1000) and secondary antibodies (HRP-conjugated, 1:5000). The bands were visualized using the Western Lighting kit (Perkin Elmer, Shelton, CT, USA) and were quantified with an Odyssey imaging system (Li-Cor, Lincoln, NE, USA).

Cell Viability Assay. Cell viability was assessed using the MTT assay (Sigma-Aldrich). The cells were incubated with 1 mg/mL MTT for 30 min to measure mitochondrial reduction of MTT to formazan. The resulting formazan crystals were solubilized in 100 μL of 2-propanol, and absorbance was measured at 560 nm and 620 nm using a NOVOstar multi-plate reader (BMG Labtech, Ortenberg, Germany).

Data Analysis. Data were analyzed using GraphPad Prism 6 Software. Normality was assessed with the Kolmogorov–Smirnov or Shapiro–Wilk test. Statistical significance was determined using the unpaired Student’s t-test for two groups or One-way ANOVA with Dunnett’s multiple comparisons for multiple groups. The results are expressed as mean ± SEM, with n indicating independent replicates. Significance levels were noted as * *p* < 0.05, ** *p* < 0.01, *** *p* < 0.001 vs. control, ns: not significant.

## Figures and Tables

**Table 1:** Docking score parameters for the interaction of *Gelsemium* indole alkaloids with the catalytic site of transglutaminase 2. The best docking scores (ΔG binding values) are presented for each alkaloid and for Z-DON. Conserved residues interacting with both Z-DON and Gelsemium alkaloids are underlined. Stereoisomeric structures for gelsemine and gelsevirine were also included.

Compound	Docking Score	∆G Binding (kcal/mol)	Binding Site Residues (4Å Distance)
Gelsemine (1′R,2′S,3S,5′S, 6′S,8′R,11′S)	−2.911	−32.24	R296, W341, Y351, E352, G353, W354, P375, V376, R377, K380, H441, T442
Gelsemine (1′*R*,2′*R*,3*R*,5′*S*,6′*S*,8′*R*,11′*S*)	−2.650	−33.19	W341, E352, G353, W354, V376, R377, K380, T442
Koumine	−2.511	−31.35	W341, G350, E352, G353, W354, V376, R377, K380, T442
Gelsevirine (1R,2S,5S,6S,7S,8R, 11S)	−3.964	−22.07	L150, E155, Y159, R296, W341, W354, V431, E437, I439, H441, T442
Gelsevirine (1R,5S,6S,7S,8S)	−3.514	−40.72	W341, Y351, E352, G353, W354, Q355, P375, V376, R377, K380, T442
Gelsevirine (1R,2S,5S,6R,7S,8R, 11S)	−1.237	−41.50	R296, E339, W341, G350, Y351, E352, G353, W354, Q355, P375, V376, R377, K380, T442
Gelsenicine	−2.204	−21.77	R296, E339, W341, G350, Y351, E352, G353, W354, Q355, K380, I439, T442
Z-DON	−2.095	−46.99	L150, D151, S152, E153, E154, Y159, R296, E339, W341, G350, Y351, E352, G353, W354, Q355, P375, V376, R377, A378, S430, V431, R433, E437, I439, T442

## Data Availability

The data used to support the findings of this study are already incorporated within this manuscript.

## Abbreviations

The following abbreviations are used in this manuscript:

Aβ	β-amyloid peptide
AD	Alzheimer’s disease
TG2	Transglutaminase type 2
TNF-α	Tumor necrosis factor-alpha
EGFR	Epidermal growth factor recepto
CDK2	Cyclin-dependent kinase 2
MAPK3	Mitogen-activated protein kinase 3
MMGBSA	Molecular Mechanics/Generalized Born Surface Area
ΔG	Gibbs free energy change
Z-DON	6-diazo-5-oxo-norleucine tetrapeptide, TG2 Inhibitor
